# More and Better Information to Tackle HIV Epidemics: Towards Improved HIV Incidence Assays

**DOI:** 10.1371/journal.pmed.1001045

**Published:** 2011-06-14

**Authors:** Incidence Assay Critical Path Working Group

## Abstract

Timothy Hallett and colleagues from the Incidence Assay Critical Path Working Group argue that a quick, easy, valid, and precise method of estimating HIV incidence in populations is needed, and discuss several new technologies to address this need.

**Summary Points**

- Investments in HIV prevention activities aiming to reduce incidence could be targeted more effectively and efficiently to successful programmes if a quick, easy, valid, and precise method of estimating incidence in populations were available.
- Laboratory methods for identifying recent infections have shown promise for this application, although their accuracy has been questioned, and progress and investment in this field has been challenged by technical and market-related issues.
- A number of activities are now underway to address these factors and several promising new technologies are anticipated in the next few years.

To stop the spread of any epidemic, it is essential to know where and among whom the infection is spreading and the impact of potential interventions. Thus, accurate information on the incidence rate of HIV—the rate of new infections in a population—could substantially strengthen and increase the efficiency of the response to the global HIV epidemic. A method to quickly and reliably estimate the incidence of HIV would have valuable applications in surveillance, programme planning, impact evaluation, and prevention trial planning, and could potentially provide an end point in community- or individual-based intervention trials (Box 1). Longitudinal cohorts (observing how many uninfected individual become infected in a set period of follow-up), mathematical models, and HIV prevalence (the proportion of individuals living with infection) measures, which have been used to estimate or approximate incidence in the past, have significant limitations for these applications [1],[2]. However, a laboratory assay (or algorithm of multiple assays) that can measure a well-characterised biomarker for the recentness of infection could be used in a single cross-sectional survey to measure HIV incidence. A “first generation” of assays of this type have been available for more than a decade [3]–[5] (recently reviewed by Murphy and Parry [6], Mastro et al. [7], and a WHO technical working group in Busch et al. [8]). However, because the estimates of incidence produced by them conflicted with other estimates and epidemiological information [9]–[14], their accuracy has been questioned.

**Box 1. Who Needs a Reliable HIV Incidence Assay?**

**Area:** ***Impact evaluation***

**Function:** To determine the impact of large-scale interventions on the rate of new infections.

**Users:** Implementers, donors/funders, researchers, advocates.

**Area:** ***Surveillance***

**Function:** To monitor transmission patterns, identify at-risk groups, and detect emerging trends in epidemic.

**Users:** Ministries of health, donors/funders, advocates.

**Area:** ***Programme and resource planning***

**Function:** To optimally target interventions and to plan for future service requirements (e.g., treatment slots).

**Users:** Programme planners, ministries of health, advocates.

**Area:** ***Trials***

**Function:** To estimate pre-trial incidence to inform trial planning (required sample size, trial length, etc.), and as an end point in community-based effectiveness trials.

**Users:** Clinical trial/research, organizations, funders/donors, researchers.

Our goal is to ensure that in the future, when an estimate of HIV incidence in any population is needed, a standard, accurate, inexpensive, and easy-to-use kit can be purchased commercially just as easily as HIV tests can be acquired to estimate HIV prevalence. This would mean that HIV incidence estimation could become routine, permitting robust, up-to-date empirical information on the trajectory and focus of the epidemic and on the impact of scaled interventions, and, in combination with other epidemiological and programme implementation information [15],[16], this would have the potential to directly influence the programmatic decisions of major implementers, funders, and donors. In September 2010, representatives from major stakeholder organizations met to review the issues and establish a strategy for achieving this goal. Major stakeholders with diverse perspectives were identified as key participants—these included the US President's Emergency Plan for AIDS Relief and the Global Fund (that need to measure impact of programmes), the Joint United Nations Programme on HIV/AIDS (UNAIDS) (tasked with tracking the course of the epidemics), the US Centers for Disease Control and Prevention (CDC) and World Health Organization (WHO) (that will produce normative guidance of the use of assays), the US National Institutes of Health (NIH) (where research on prevention intervention evaluation is reliant on incidence measurement, and which can support research into new technologies), and diagnostics manufacturers (many of which have not entered the market for incidence assays). In this article, we summarise their analysis and the strategy that was agreed upon (a fuller briefing document is provided in Text S1).

## Current Status of Laboratory-Based Assays

The principle of a test for recent infection is to measure a biological target (“biomarker”) that is related to an early phase of HIV infection (e.g., antibody concentration, proportion, avidity, etc.). However, many such biomarkers are associated with a very large between-individual variation and this makes it difficult to characterise the fundamental test characteristics (Box 2). This variation can mean that a proportion of individuals with long-standing infection test as “recently” infected. The proportion of chronically infected individuals that are misclassified as recent—termed the false recent rate (FRR)—has been found to vary widely between populations; from 0.8% in south Vietnam [17] to 16% in Uganda (before additional screening using behavioural, immunologic, or virologic information) [11],[12],[14],[18]. This can be due to differences in HIV subtype, epidemic phases, different levels of total IgG in different populations, and the extent of antiretroviral treatment use [10],[19],[20]. Nevertheless, if the local and current FRR is known accurately, it can be factored into the results of the assay to obtain an unbiased incidence estimate [13],[21]–[23]. However, because the measurement of the FRR is itself a substantial undertaking (since it requires finding a large and representative sample of individuals with HIV known *not* to have been infected recently), this has not often been done. In a review of 39 studies that measured HIV incidence using BED assays, most did not account for any misclassification [24], and among those studies that did assume a non-zero FRR, the values used were not measured in the local populations but instead were taken from previous studies in different populations [24]. This leads to substantial errors in the absolute level of incidence estimated and the pattern with respect to time and age [11]–[14],[19],[24]–[27].

**Box 2. Properties of an HIV Incidence Assay**

*Incidence Test Metrics*

Incidence assays are used to estimate HIV incidence because they measure a well-characterised biomarker for the recentness of infection. In the ideal case, the assay works so that one result (“test-positive”) is returned for a period early in infection and at other times a different result (“test-negative”) is returned. The average time it takes for individuals to progress from the test-positive to the test-negative state is called the mean duration, 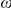 (see Figure 2). Incidence can thus be estimated as 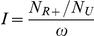, where 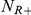 is the number test-positive and 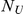 is the number not infected, and this formula is similar to the classic epidemiological calculation, “Prevalence = Incidence×Duration” [22],[23].

However, in practise, a subset of individuals in a population may not ever progress to the test-negative stage, and some individuals that had progressed to the test-negative state may later regress to the test-positive state. The proportion of chronically infected individuals with a test positive result in a particular population at a particular time can be measured (among a representative sample of infected individuals known not to have been infected recently) and is termed the false recent rate (FRR), 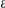 (see Figure 2). Note that this parameter is not equivalent to the traditional definitions of test “specificity” or “negative predictive value”.

The updated statistical estimator that allows an unbiased estimate of HIV incidence using an imperfect test (i.e., an FRR greater than zero) is 
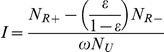
, where 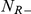 is the number of test-negative results [22]. But this estimator only works if the test characteristics (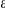 and 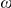) are accurately and precisely measured for that particular population at that particular time (both these parameters can vary substantially between populations and over time [8],[11],[18],[37]). If the estimate for these calibration parameters is not accurate, there will be substantial bias in the estimate of incidence [12],[13],[19]; and if they are not precisely estimated, then incidence estimates will be uncertain even if the cross-sectional population survey is large. Critically, the mean duration and the FRR operate as a pair of parameters that jointly specify the test characteristics, so in any calculation, the way in which those parameters are evaluated must be the same.

For many applications of the first generation assays, these test characteristics have not been measured accurately for the population in which the test is being used, and this is the reason that published assay-derived estimates have been inaccurate [24] (see Figure 2).

Another issue is the large sample sizes required for precise incidence estimates using incidence assays. Sample sizes to record incidence are inevitably larger than those required to gain the same precision in estimates of prevalence because incident infections are approximately 10-fold less common than prevalent infections. But, with current incidence assays, the need for large sample sizes is compounded by both the necessity to account for the misclassification rate and the uncertainty in the characteristics of the assay itself. For instance, to reach an 80% chance of recording a statistically significant change if incidence was really reduced by half in South Africa would require two surveys each of 6,000 adults if FRR = 0%, but 15,000 adults per survey if FRR = 5% [23]. In other settings with lower baseline incidence, or with more modest reductions in incidence, sample sizes would have to be even greater.

Encouragingly, a new generation of incidence assays that are based on different biomarkers is now in development [28]–[31]. In early tests, the FRR for one avidity assay [32] was as low as 1% [33]. There are also promising preliminary data that algorithms (combinations of several assays) can generate very low FRR values (e.g., BED and a particular Bio-Rad avidity assay in specimens from the US has FRR = 0.8% [33]). This performance level, if reproduced in other populations (and it is not certain that they will), could be sufficient for reliable incidence estimates across populations in surveys of feasible sample sizes.

## Challenges That Must Be Addressed

To plan the next steps, we have tried to identify the factors that have challenged progress in recent years. These include the following:

1. There has been a need for guidance and support for developers of incidence assays. No normative agency or scientific body has developed performance standards that incidence assays must meet and, because incidence assays are for “population use” rather than individual diagnosis, traditional regulatory regimes used by the US Food and Drug Administration (and other regulatory agencies) to evaluate and approve diagnostic assays have not been automatically required. In the absence of guidance from a scientific body or normative agency, many assay developers have endeavoured to evaluate their candidate assays with any of the very few and not fully representative seroconversion panels (collections of samples taken frequently from newly infected individuals) that were readily accessible. The incompleteness of these evaluations makes it hard to identify the best assays, anticipate their performance at estimating incidence in real populations, or compare different assays. Development of assays by companies has been impeded because they typically only have access to the small subset of all possible specimens that have been collected and are available commercially.
2. There has been a need for clear guidance for the users of incidence assays. Many assays are in use in surveillance systems in Europe, but there has been no guide as to the relative strengths of each approach [6], and for the most widely used assay (the BED), the CDC and UNAIDS had adopted different positions on whether or not it could be used routinely [34],[35]. The absence of a clear consensus from major public health agencies involved in HIV prevention has led users to apply assays inconsistently.
3. There has been a lack of market incentive for manufacturers to invest in developing incidence assays. It has been estimated that the global demand for HIV incidence assays could be as high as several million over 5 years, but it could be as low as just a few hundred thousand [36]. This relatively small and uncertain market size is a significant obstacle to investment. Another deterrent has been the apparent lack of consensus among public health agencies about assay performance requirements and under what circumstances an assay would be recommended.

## Next Steps in the Critical Path

**Figure 1:**
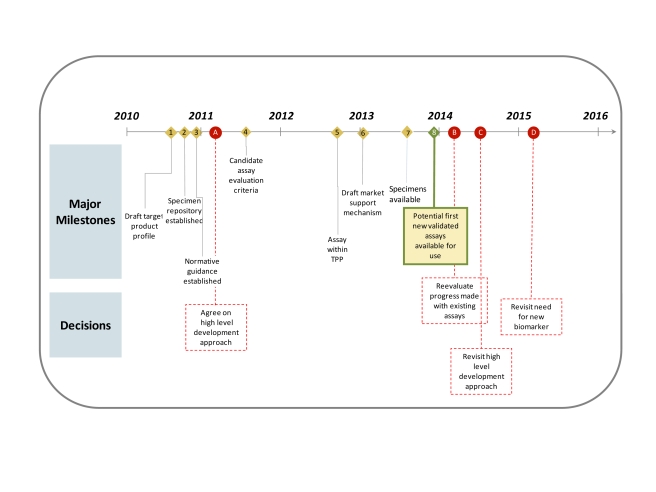
Key milestones and decision points in the critical path over the next 5 years. Anticipated timing of major milestones (numbered diamonds with solid lines) and key decisions (red circles with dashed lines) over the next 5 years in the critical path.

To tackle those challenges and move towards the goal of having a thoroughly validated incidence assay, we have charted the activities, milestones, and decisions that will be required in the coming years (Figure 1). This analysis suggests that the first validated assays (or algorithms) could be available for use by the end of 2013.

In the critical path, attention should first be devoted to understanding the target product profile. This is a list of technical specifications for the assay that meets the performance requirements of intended users (see Text S1). We propose that an assay with an FRR that can be measured accurately for the target population and is confidently not more than 2% and a mean duration of between 4 and 12 months, is a reasonable *acceptable* target. Other key properties include storage conditions, shelf life, and sample type.

Secondly, a repository of specimens will be established to aggregate material from a wide range of different populations (including seroconverter cohorts, repeat blood donors, and samples of individuals with longstanding infection), viral clades, sample types (including plasma, serum, and dried blood spot), and epidemic setting. This resource would be essential for a systematic evaluation and calibration of existing incidence assays and to support development of new assays. It would also provide insights into how an algorithm of incidence assays could be assembled to provide better results. Providing industry with access to the panels for early feasibility testing and evaluation would reduce one of the barriers to their greater involvement. The Health Protection Agency in the United Kingdom will now establish such a repository and it is anticipated that a significant number of samples will have been received by mid-2011 and that data on the characteristics of existing assays (and algorithms using them) will be available in mid-2012. At this point, it is hoped that there will be evidence of at least one assay meeting the target product profile.

Next, the assays must undergo validation—that is, their incidence estimates compared to other epidemiological information such as measurements of incidence from longitudinal cohorts. These validation studies should ideally take place in a range of communities (with different epidemic phase, clade, antiretroviral therapy use, and population type). This may be achieved in partnership with existing cohort studies that have archived specimens.

After validation, the normative guidelines on incidence assays, reflecting which assays have been fully evaluated and validated, will be updated and publicised regularly. This should include a standard method for analysing data from incidence assays and reporting results (including quantifying the uncertainty in incidence estimates).

From 2013, the priority would shift to supporting the scaled, quality-assured manufacture of the assay. This support may take the form of a purchase agreement, for which the purchase of the tests is guaranteed, reducing the risk for the supplier. Preliminary discussions on the feasibility and potential form of such agreements have now begun between manufacturers, donors, and funders.

In parallel, there should also be investment in the development of new biomarkers for improved assays in the medium to long term, and NIH has issued a program announcement (PA-10-212) for proposals with this aim. Examples of new possibilities for biomarkers include cytokine profiles and within-individual viral diversity measures, although it is too early to tell if a usable and reliable assay could be created to measure them.

## Conclusions

Progress in recent years towards the use of a robust and well characterised incidence assay has been challenged by several factors but many of these can now be overcome. A new generation of incidence assays promises to be more accurate, but the appropriate adoption and implementation of these new tests requires close alignment of evaluation, validation, and commercialisation activities. We believe that if this is achieved, and the information is meaningfully triangulated with other epidemiological and programme implementation information [15],[16], then the effectiveness and efficiency of our response to the HIV epidemic will be enhanced, and this will be to the benefit of all those that remain at risk of HIV infection.

**Figure 2:**
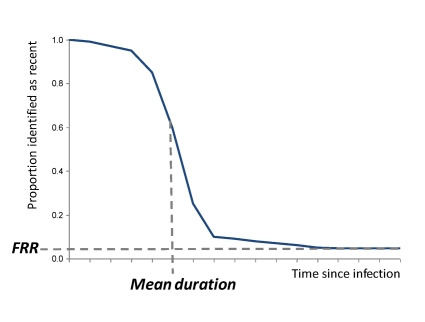
Proportion of individuals “test positive” on the incidence assay versus time since infection. The two main parameters (mean duration and FRR) are indicated under the simplifying assumption that individuals do not progress (this assumption is not made in the statistical equations but is made here only to simplify the graphic presentation).

## Supporting Information

Text S1 Full-length version of briefing paper.
(DOCX)

## Abbreviations

CDC: US Centers for Disease Control and Prevention
FRR: false recent rate
NIH: US National Institutes of Health
UNAIDS: Joint United Nations Programme on HIV/AIDS
WHO: World Health Organization
