# Correction: More and Better Information to Tackle HIV Epidemics: Towards Improved HIV Incidence Assays

**DOI:** 10.1371/annotation/0931760c-95fa-441c-a797-7b410a4c9326

**Published:** 2011-07-20

**Authors:** Incidence Assay Critical Path Working Group

Timothy Mastro, Family Health International, should be listed as a Member of the Incidence Assay Critical Path Working Group. 

