# Supplementary material for: More and Better Information to Tackle HIV Epidemics: Towards Improved HIV Incidence Assays
Source: PLoS Med. 2011 Jun 14;8(6):e1001045. doi: 10.1371/journal.pmed.1001045 (PMC3114871; doi:10.1371/journal.pmed.1001045)
Supplement: Text S1 — Full-length version of briefing paper. (DOCX) [file pmed.1001045.s001.docx]

***More and Better Information to Tackle HIV Epidemics: Towards Improved HIV Incidence Assays***

***Incidence Assay Critical Path Working Group***

**Text S1: Full length version of Briefing Paper**

**A reliable measure of HIV incidence, the rate of new HIV infections in a population, is urgently needed. Currently, spending of 11 billion dollars needed annually for HIV prevention programmes and the evaluation of existing interventions is guided by estimates and approximations of incidence that suffer from bias and substantial uncertainty. Investments in HIV prevention activities aiming to reduce incidence could be targeted more effectively and efficiently to successful programmes if a valid and precise incidence assay or algorithm were available. A number of laboratory measures of HIV incidence have been developed since the mid-1990s, but these “first generation” assays often generate estimates that are inconsistent with other epidemiological information. Most activity in this area has been in the public sector, as the commercial sector has not perceived HIV incidence assays to be a large enough market to justify investment. Fortunately, a number of activities are now underway to address these challenges and several promising new technologies are anticipated in the next few years. However, the overall effort has suffered from limited coordination and common strategic purpose. In this article, we describe the situation and chart a course toward the routine use of reliable HIV incidence assays in the coming years.**

To stop the spread of the Human Immunodeficiency Virus (HIV), it is essential to know where and among whom HIV is spreading and to assess the potential impact of various prevention interventions. Thus, accurate information on the incidence of HIV – the rate of new infections in a population – could substantially strengthen and increase the efficiency of the response to the epidemic as well as increase the sustainability of funding. A method to quickly and reliably estimate the incidence of HIV would have valuable applications in surveillance, programme planning, impact evaluation, and prevention trial planning, and could potentially provide an end-point in community or individual-based intervention trials (Box 1). One way to achieve this would be via a laboratory assay (or algorithm of multiple assays^[[1]](#footnote-2)^) that can measure a well-characterized biomarker for the recentness of infection. Thus, a single cross-sectional survey with a well calibrated ‘incidence assay’ could be used to estimate the current rate of new HIV infections in the population. A “first-generation” of assays of this type have been available for more than a decade ([1-3](#_ENREF_1)) (recently reviewed by Murphy and Parry ([4](#_ENREF_4)), Mastro *et al.* ([5](#_ENREF_5)), and a WHO technical working group in Busch *et al.* ([6](#_ENREF_6))). However, because the estimates of incidence produced by them conflicted with other estimates and epidemiological information ([7-12](#_ENREF_7)), their accuracy has been questioned.

In September 2010, representatives from major stakeholder organizations met to discuss the current options for estimating incidence, establish a vision for the future and develop a pathway for achieving this version. It was agreed that we urgently need a standard, accurate, inexpensive, and easy-to-use commercial kit that can be used to estimate HIV incidence as easily as current tests enable us to determine HIV prevalence. In this article, we describe the common vision that emerged at this meeting and the consensus strategy that was developed to achieve it.

***The Need for an Incidence Assay***

A number of methods are currently being used to estimate HIV incidence, but each of these approaches has significant limitations. In population surveillance, mathematical model estimates of incidence, based on national/sub-national HIV prevalence data, have often served as a substitute for actual HIV incidence measurements. However, HIV prevalence is related to HIV incidence in a complex fashion, especially in mature epidemics, and because most untreated individuals spend a decade living with HIV after infection ([13](#_ENREF_13)), changes in incidence based on prevalence data can only be detected retrospectively several years after the changes have taken place ([14](#_ENREF_14)). As an example, up to five years after PEPFAR and the Global Fund began prevention activities, HIV prevalence data could show little evidence of any impact even if the programmes were truly highly effective ([15](#_ENREF_15)). The connection between prevalence and incidence will become even weaker with the increasing (but uneven) availability of antiretroviral treatment and it is anticipated that prevalence will rise as those infected with HIV are able to live longer, potentially masking decreases in incidence that are a result of successful interventions. Targeting prevention efforts based on HIV prevalence (rather than incidence measurement) could lead to resources directed to communities with lower rates of new infection ([16](#_ENREF_16)); or to missing opportunities to target some at-risk demographic groups (e.g. at older ages, low prevalence rates can hide an increase in incidence at those ages that would represent a valuable point of intervention ([17](#_ENREF_17), [18](#_ENREF_18))). Trends in prevalence among young people can provide a proxy for trends in incidence, but this only informs about that age group. Moreover, the lower prevalence rates in this group require larger sample sizes to measure prevalence precisely, and, if measured among pregnant women attending antenatal clinics, trends can be distorted by changes in likelihood of being included in surveillance (e.g. changes in fertility or pre-natal care), and the rate of change of prevalence in a particular age group is still slow compared to real changes in incidence.

In observational research studies and in community-based prevention trials, HIV incidence has been measured using longitudinal cohorts, in which individuals are tracked over several years. However, these studies are very expensive and complex to run and it has become clear that enrolling individuals in longitudinal cohorts can substantially affect their risk behaviour, due in part to the observation *per se* (the classically described "Hawthorne effect” ([19](#_ENREF_19))), and to the provision of high standard prevention measures that reduce risk. For instance, NIH recommends that persons in control arms of prevention trials be provided with counselling and access to proven methods for reducing the chances of acquiring HIV, such as condom use and male circumcision. There is also the possibility of a enrolment and retention biases, whereby those who are uninfected and enter the study are not representative of the wider population and loss to follow-up is such that those who remain in the cohort may not be representative of those initially recruited. These biases effectively limit the internal and external validity of derived measures ([20](#_ENREF_20)). For instance, in three years of follow-up of a cohort of sex workers in Kenya, reported condom use increased steadily with follow-up, and incidence of HIV in the cohort (and sexually transmitted infections) declined to a small fraction of the initial rate ([21](#_ENREF_21)), but it is unlikely that this change was representative of the broader population of sex workers.

Rates of new HIV cases reported have sometimes been used to make inferences about the trajectory of the epidemic ([22](#_ENREF_22), [23](#_ENREF_23)). However, trends in diagnosis and reporting will be related to incidence through an unknown interval between infection and diagnosis that is likely to change over time, especially in the era of increased testing, antenatal screening programmes and the availability of treatment.

Without a reliable way to rapidly and accurately estimate HIV incidence, the field is left with critical knowledge gaps or uncertainties that limit the effectiveness and efficiency of the response to the epidemic. The absence of clear-cut evidence for the impact of major programmes may even weaken the case for continued investment. Eleven billion dollars are estimated to be needed for HIV prevention in 2010 ([24](#_ENREF_24)), but major international donors, including PEPFAR and the Global Fund, have so far been unable to definitively gauge the impact of their previous investments. The “Know your epidemic, know your response” framework ([25](#_ENREF_25), [26](#_ENREF_26)) aims to direct programmatic support to those populations most at risk, but because the incidence rates used come from mathematical models rather than on empirical incidence data, it can still be unclear where to direct support.

Given the demand for incidence information and the limitations of existing epidemiological methods, a method to estimate the incidence rate in a population from a single cross-sectional survey has many appealing advantages: it immediately provides an up-to-date reading on the trajectory of the epidemic, the incidence rate is ascertained at the first instance an individual is contacted (maintaining external validity), and follow-up of individuals is not required (as would be in a cohort study)—substantially reducing costs and eliminating exposure to recruitment and retention biases. Such an assay would share some characteristics with a highly sensitive diagnostic that could identify very recently infected individuals (See Box 2). However, the focus of this current effort is for a standardized tool for measuring the HIV incidence rate at the population level.

***Current Status of Laboratory-Based Assays***

Since their introduction in the mid-1990s ([1](#_ENREF_1)) the use of laboratory-based methods to measure incidence in cross-sectional surveys has been limited by a number of technical challenges. The principle of a test for recent infection is to measure a biological target (“biomarker”) that is related to an early phase of HIV infection (e.g., antibody concentration, proportion, avidity, etc.). Many such biomarkers are associated with a very large between-individual variation, making it difficult to characterize one fundamental test characteristic—the mean duration following infection that the marker is detectable (Box 3). Unfortunately, accurate and precise knowledge of this duration is pivotal when using data from cross-sectional surveys to estimate incidence

The range of responses to early infection also varies for different HIV subtypes. Early approaches (such as ‘detuned’ assays ([3](#_ENREF_3))) evaluated only subtype B antigens; subsequently between-individual variability was found to be even greater for other subtypes. The “BED test” (BED IgG-Capture Enzyme Immunoassay) addressed this by using antigens from subtypes B, E, and D, and its response is more uniform across subtypes ([27](#_ENREF_27)). In part for this reason, the BED was made commercially available in 2005 and became the most widely used product for HIV incidence estimation.

A further challenge is that assays can misclassify a proportion of individuals with long-standing infection as ‘recently’ infected, rather than as chronically infected. Importantly, this phenomenon could not have been detected from original analyses of specimens from seroconverter panels infected for a short time. Although misclassification has been repeatedly shown for the BED assay, it probably occurs for many other types of assays too. If this is ignored, the resulting HIV incidence estimate can be too high, especially in high prevalence populations, where the number misclassified overwhelms the number of those truly recently infected ([9-12](#_ENREF_9), [28](#_ENREF_28)).

The proportion of chronically infected individuals that are misclassified as recent – termed the “False Recent Rate” (FRR) (Box 3) – has been found to vary between populations; from 0.8% in south Vietnam ([29](#_ENREF_29)), to 1.8% in South Africa and up to 16% in Uganda^[[2]](#footnote-3)^ ([9](#_ENREF_9), [10](#_ENREF_10), [12](#_ENREF_12), [30](#_ENREF_30)). This can be due to differences in HIV subtype, epidemic phases, different levels of total IgG in different populations, and the extent of antiretroviral treatment use ([8](#_ENREF_8), [31](#_ENREF_31), [32](#_ENREF_32)). But, if the local and current FRR is known accurately, it can be factored into the results of the assay to obtain an unbiased incidence estimate ([11](#_ENREF_11), [33-35](#_ENREF_33)). However, because the measurement of the FRR is itself a substantial undertaking (since it requires finding a large and representative sample of HIV-infected individuals known *not* to have been infected recently), this has not often been done. In a review of 39 studies that measured HIV incidence using BED assays, for instance, most did not account for any misclassification ([28](#_ENREF_28)). Among those studies that did assume a non-zero FRR, the test characteristics used to assess this were not measured from the local population, but instead were taken from previous studies in different populations ([28](#_ENREF_28)). That approach could lead to biased results despite the fact that the estimate was ‘corrected’ for misclassification ([10](#_ENREF_10), [11](#_ENREF_11), [31](#_ENREF_31)). The package insert for the BED assay, written in 2005 (before many studies of the false recent rate were done) and still in use today, does acknowledge the issue of misclassification, but does not emphasize its variability across populations, stating that: “About 2-3% of people with long-term HIV infections, including AIDS, may be misclassified.” ([36](#_ENREF_36)).

In fact, several validation studies have shown that BED–based HIV incidence estimates are too high. In 20 published estimates of HIV incidence using results from BED assays that could be directly compared with data from a reference incidence rate, the estimates from the BED assay were, on average, 27% higher, and, in some extreme cases, more than 400% higher^[[3]](#footnote-4)^ ([9](#_ENREF_9)). Within-population patterns of BED-derived incidence can also be at odds with other information, such as the older ages of peak incidence recorded in the BED estimates for Uganda ([37](#_ENREF_37)), when compared to cohort and modelling data ([38-40](#_ENREF_38)).

A further issue is the large sample size requirement for incidence estimates using laboratory assays. Sample sizes to record incidence are inevitably larger than those required to gain the same precision in estimates of prevalence simply because incident infections are approximately ten-fold less common than prevalent infections. But, with current ‘first generation’ incidence assays, the need for large sample sizes is compounded by both the necessity to account for the misclassification rate and the uncertainty in the characteristics of the assay itself (even if the estimate is perfectly accurate for the population being studied). For instance^[[4]](#footnote-5)^, to reach an eighty percent chance of recording a statistically significant change if incidence was really reduced by half in South Africa would require two surveys each of 6,000 adults if FRR=0%, but 15,000 adults per survey if FRR=5%. In other settings with lower baseline incidence, or with more modest reductions in incidence, sample sizes would have to be even greater.

Another challenge for the field has been the lack of a clear consensus on the way in which to analyze incidence data. Ten different statistical formulae have been used to produce incidence estimates that, when applied to exactly the same data, produce different estimates (especially when comparing the older estimator to the more recent ones that have some facility to reflect misclassification) ([28](#_ENREF_28)). In most published studies, the reported confidence intervals around incidence estimates have been based only on the survey sample size, rather than also accounting for uncertainty in assay characteristics (see Box 3) ([28](#_ENREF_28)). Figure 1 illustrates the confidence intervals that would be calculated with and without incorporating uncertainty in test characteristics and highlights that when these parameters are not considered incidence estimates appear to be much more precise than is actually the case. These important effects could lead to spurious conclusions about the effectiveness of an intervention or the overall trend of the HIV epidemic. Attention is drawn to these effects in new or forthcoming WHO^[[5]](#footnote-6)^ and OGAC^[[6]](#footnote-7)^ guidance documentation. Still, a highly visible debate continues about the optimal way to statistically analyze assay data ([11](#_ENREF_11), [34](#_ENREF_34), [35](#_ENREF_35), [41-45](#_ENREF_41)), although these exchanges have probably obscured the more important consensus among statisticians; the issue of fundamentally marrying the actual test characteristics with the assumptions made about them. A related issue is the need for a standardized method for estimating the assays characteristics (see Box 3); whereas several different methods have been proposed for doing this, the optimal form of the analysis is not clear and because small differences in estimates of tests characteristics translate into larger difference in the estimates of incidence, it will be important to reach agreement on the best way to make these measurements.

Encouragingly, a new generation of incidence assays are now in development ([46-49](#_ENREF_46)). These are based on different immunologic principles — notably, antibody avidity, which may be more tightly linked to the recentness of HIV infection than antibody concentration or proportions (upon which earlier incidence assays have been based). In early results using specimens from chronically infected individuals in the US (ALIVE Cohort), the false recent rate for one avidity assay ([50](#_ENREF_50)) was as low as 1% ([51](#_ENREF_51)). This performance level, if reproduced in other populations, could be sufficient for reliable incidence estimates across populations (provided, of course, that there is appropriate statistical treatment of the test characteristics (Box 3) and a sufficiently large sample size ([35](#_ENREF_35))). Furthermore, there are promising preliminary data that algorithms (combinations of several assays) can generate very low FRR values. For example, the combination of BED and a particular *BioRad* avidity assay (and other virologic and immunological markers) in clade B specimens from the US resulted in an FRR as low as 0.8% ([51](#_ENREF_51)). However, we note that these early findings should be treated with caution, since it is not known whether this performance could be reproduced with other clades or populations, which will require a more systematic investigation of the assay (as described below).

***Challenges That Must Be Addressed***

To plan the next steps, it is helpful to reflect on those factors that have challenged progress with the first generation of assays. Building on the findings of a technical working group of the WHO ([6](#_ENREF_6)), we have highlighted the following four issues:

1) There has been a need for guidance for developers of incidence assays. No normative agency or scientific body has yet developed performance standards that incidence assays must meet. Further, because incidence assays are for “population use” rather than individual diagnosis (the between-individual variability reduces the predictive value of each individual’s test result: see Box 2), traditional regulatory regimes used by the FDA (and other regulatory agencies) to evaluate and approve diagnostic assays have not been automatically required for these assays. In the absence of guidance from a scientific body or normative agency, many assay developers have endeavoured to evaluate their candidate assays with any of the very few and not fully representative seroconversion panels (collections of samples taken frequently from newly infected individuals) that were readily accessible or commercially available. The incompleteness of these evaluations makes it hard to fully characterize or compare the performance of the different assays. For example, the results obtained for an assay evaluated with subtype B seroconversion panels cannot be compared with those obtained for an assay evaluated with subtype C seroconversion panels. Furthermore, good performance of an assay on a restricted set of specimens does not guarantee robust estimation of incidence in large, real world populations (where rare responses can introduce substantial biases in the estimate). So some assays have become widely adopted without the degree of evaluation and validation that has since been recognized as important. The WHO technical working group has begun work on a draft of guidance for assay developers and in the coming months, a secretariat will be established to monitor consistency.

2) For the thorough evaluations that are required, there is a need for a centralized repository of well-characterized specimens, from both seroconverters and chronically infected individuals. This repository would serve to help product development efforts, and provide a standardized way for the performance of new incidence assays to be definitively evaluated. A ‘virtual repository’ (list of available specimens) has been compiled by the WHO technical working group, which has highlighted that these specimens are enormously expensive to collect, and existing specimen sets are rare and difficult to obtain. Nevertheless, collecting, characterizing, and storing the specimens, although logistically complex and expensive, will be necessary for rigorous evaluation of assays. Having the specimens, or a subset thereof, made available to developers (as “feasibility” panels) may also make it easier for companies that do not have access to specimen sets to enter the field. A standard procedure for pre-testing may be required before specimens from the repository are used (to avoid unnecessary waste), the nature of which could be agreed upon by a steering group or secretariat*.* Ideally, the repository would store specimens from a comprehensive range of clade, sample types (dried blood spot, plasma, etc.), and population type, from both seroconverter panels and chronically infected populations.

3) There has been a need for clear guidance for the users of incidence assays. CDC and UNAIDS have adopted different positions on how to use of the BED assay ([52](#_ENREF_52), [53](#_ENREF_53)). Moreover, there are many different assays in regular use besides BED ([4](#_ENREF_4)), especially in Europe, for which no guidance has been issued about how to use and compare results. The absence of a clear consensus from major public health agencies involved in HIV prevention has led users to apply assays inconsistently. Guidance on the appropriate use of incidence assays is essential to indicate when particular technologies can confidently be used and when not. Recently, the WHO technical working group completed draft guidelines that address these issues; OGAC has released similar guidance, representing a valuable step in harmonizing guidance for users.

4) There has been a lack of market incentive for manufacturers to invest in developing incidence assays. It has been estimated that the global demand for HIV incidence assays could be as high as several million over five years, but it could be as low as just a few hundred thousand ([54](#_ENREF_54)). This relatively small and uncertain market size is a significant obstacle to investment because assay development can be an expensive process that involves many stages, including research, validation, product industrialization, manufacturing, distribution, quality control, and post-marketing surveillance. In addition, the lack of apparent consensus among public health agencies about assay performance requirements and under what circumstances it would eventually be used further reduces the attractiveness to companies of new investment in this area.

***Next Steps in the Critical Path***

Our goal is to ensure that in the future, when an estimate of HIV incidence in any population is needed, a standard, accurate, inexpensive and easy-to-use kit can be purchased commercially, just as easily as HIV tests can be acquired to estimate HIV prevalence. This would mean that HIV incidence estimation could become routine, permitting robust, up-to-date empirical information on the impact of scaled interventions and the trajectory and focus of the epidemic, and this information would have the potential to directly influence the programmatic decisions of PEPFAR and the Global Fund in a timely way. This is our ‘*vision of success*’ and it is most likely to be reached by a series of key actions and decision points that describe a ‘*critical path*’. Along this path there are several underlying milestones to be achieved by different constituencies:

(i) A resource for supporting research and evaluating and validating assays (with reliable sources for well-characterized specimens), providing a clear route for developers to enter the field;

(ii) An incidence assay (or combination of assays in an algorithm) with well-defined, robust performance characteristics;

(iii) Evidence that the assay can be used to reliably estimate HIV incidence in a range of populations (based on comparisons with other epidemiological information and measurements);

(iv) A commercial manufacturer with an incentive to sustain a high quality supply; and

(v) Strong guidance for users, including consensus standards on the statistical methods for estimating incidence in populations (and estimating test characteristics).

The anticipated timing of major milestones and key decisions along the critical path are shown in Figure 2. This analysis suggests that the first validated assays (or algorithms) could be available for use by the end of 2013.

In the critical path, attention should first be devoted to understanding the target product profile. This is a list of *technical* specifications for the assay that meets the performance requirements (acceptable and ideal) of intended users, and is defined jointly by users, normative agencies, developers, and funders (Box 4). Although the balance of specifications is complicated by the differences in HIV subtypes and the performance of an incidence assay in different populations/settings (due to epidemic phase, ART frequency etc), insights from two years of effort supported by the WHO working group indicate that an assay with an FRR that can be measured accurately for the target population and is confidently not more than 2%, and a mean duration of between 4 and 12 months, is a reasonable *acceptable* target. Other key properties include storage conditions, shelf life and sample type. In addition to these technical characteristics, there are many other considerations – most importantly, ease-of-use criteria and ultimate price to the customer – that reflect the way in which the product will be used but are not formally specified by the TPP.

Secondly, a repository of specimens will be established to aggregate material from a wide range of different populations (including seroconverter cohorts, repeat blood donors, and samples of individuals with longstanding infection), viral clades, sample types (including plasma, serum and dried blood spot) and epidemic setting. This resource would be essential to support a formal and systematic evaluation and calibration of existing incidence assays and to support development of new assays. The performance characteristics of existing assays and new assays could then be directly compared using a spectrum of appropriate specimens to fully characterize their performance across different clades, stages of infection, and populations^[[7]](#footnote-8)^. This evaluation would not only provide evidence of how any assay (even those that have received less attention) perform in particular settings but would also provide insights into how an algorithm of incidence assays could be optimally assembled to provide better results. The specimen repository would be a common resource for evaluation, and would define the global standard for evaluation of incidence assays: new assays would not be used without being evaluated with specimen panels in the repository. A grant from the Bill and Melinda Gates Foundation has recently been awarded to the Health Protection Agency in the UK to establish such a repository. It is anticipated that a significant number of samples will have been received by mid-2011 and that data on the characteristics of existing assays (and algorithms using them) will be available in mid-2012. At this point, it is hoped that there will be evidence of at least one assay meeting the target product profile.

After evaluation/calibration with samples from the specimen repository, assays must undergo validation, in which assays are compared to other epidemiological information such as a directly comparable measurement of incidence in a longitudinal cohort. These validation studies should ideally take place in a range of communities (with different epidemic phase, clade, ART use, population type). Only at this level can the usefulness of the assay for measuring incidence be fully determined. It should be anticipated that this could bring significant costs to the validation process. Cohort studies that have already reported a prospectively measured incidence rate could be approached to share archived specimens so that corresponding assay-based estimates can be produced and compared.

The same repository will be used in the evaluation of any future assay. Industry should also be actively encouraged to develop new assays (historically, most new technologies have been developed by researchers or public health agencies) and providing commercial access to the panels would reduce one of the barriers to their greater involvement.

After validation, the normative guidelines on incidence assays, reflecting which assays have been fully evaluated and validated, will be updated and publicized regularly. Major implementer such as PEPFAR and the Global Fund would begin to take advantage of the assays to evaluate programmes and sharpen insights on areas for priority investments, in line with that guidance. The priority would also shift to supporting the scaled, quality-assured manufacture of the assay. This support may take the form of a purchase agreement, for which the purchase of the tests is guaranteed, reducing the risk for the supplier. Preliminary discussions with manufacturers, donors and funders have now begun with this aim.

Given that the current generation of incidence assays and their analysis have not been sufficiently accurate for general use in all relevant global settings, and that next generation are still under development (and pending evaluation), investments in the development of new biomarkers will also be an important complimentary step toward improved assays in the medium-to-long term. Examples of new possibilities for biomarkers include cytokine profiles and within-individual viral diversity measures, although for each it is too early to tell if a usable and reliable assay could be created to measure them. There is also the possibility of a single assay platform to capture a holistic reading of the immune response (antibody titer, avidity, specific isotype and subclass antibody response) performing like a complex algorithm of techniques in an easy-to-use format ([55](#_ENREF_55)). These new approaches form the foundation for a ‘third generation’ of assays. The evaluation of these assays would also draw upon the specimen repository (provided specimens are of the appropriate types; for viral diversity validation the requirements may be different) and, to some degree, normative guidance that was developed for population-based assays.

Given the range of stakeholders involved in the critical path and the need to maintain rapid progress and make firm decisions, it will be important to involve an expert panel to provide technical and strategic oversight.

***Conclusions***

Progress in recent years towards the use of a robust and well characterized incidence assay has been challenged by several factors, many of which are now beginning to be overcome through improved dialogue between researchers, major funders/donors, normative agencies and manufactures, and we are confident this will continue in the spirit of collective ownership of this vision of success. A new generation of incidence assays promises to be more accurate, but the appropriate adoption and implementation of these new tests requires close alignment of evaluation, validation and commercialization activities. We believe that concerted and coordinated efforts among many parties - including normative bodies (UNAIDS, WHO, CDC), major implementers of interventions (PEPFAR, GFATM), funders (BMGF), scientific researchers (NIH, CDC), manufacturers, regulatory bodies, epidemiologists, and statisticians – will enable the field to navigate a critical path to the realization of our stated goals. Further, we believe that if this is achieved, the effectiveness and efficiency of our response to the HIV epidemic will be enhanced, to the benefit of all those that remain at risk of HIV infection.

***Acknowledgment***

The authors are grateful to Kaisa Sakrison and Kim Shaffer for organising the preparation of this article, and to the Bill & Melinda Gates Foundation and The Wellcome Trust for funding support.

The authors have declared that no competing interests exist.

The article draws on some material originally written as a background reading document for a meeting sponsored by the Bill & Melinda Gates Foundation and the National Institutes of Health and completed under a contract between Imperial College London and the Bill & Melinda Gates Foundation.

The first draft of the paper was written by Timothy Hallett, Peter Piot, Christine Rousseau, Bill Rodriguez, Amy Wong and Maurine Murtagh; all members of the working group contributed to drafting several further versions of this document.

***References***

1. Brookmeyer R, Quinn TC. Estimation of current human immunodeficiency virus incidence rates from a cross-sectional survey using early diagnostic tests. Am J Epidemiol. 1995 Jan 15;141(2):166-72.

2. Quinn TC, Brookmeyer R, Kline R, Shepherd M, Paranjape R, Mehendale S, et al. Feasibility of pooling sera for HIV-1 viral RNA to diagnose acute primary HIV-1 infection and estimate HIV incidence. AIDS. 2000 Dec 1;14(17):2751-7.

3. Janssen RS, Satten GA, Stramer SL, Rawal BD, O'Brien TR, Weiblen BJ, et al. New testing strategy to detect early HIV-1 infection for use in incidence estimates and for clinical and prevention purposes. JAMA. 1998 Jul 1;280(1):42-8.

4. Murphy G, Parry JV. Assays for the detection of recent infections with Human Immunodeficiency Virus Type 1. Eurosurveillance. 2008;13(17-19):4-10.

5. Mastro TD, Kim A, Hallett TB, Rehle T, Welte A, Laeyendecker O, et al. Estimating HIV Incidence in Populations Using Tests for Recent Infection: Issues, Challenges and the Way Forward Journal of HIV/AIDS Surveillance & Epidemiology. 2010;2(1):7.

6. Busch MP, Pilcher CD, Mastro TD, Kaldor J, Vercauteren G, Rodriguez W, et al. Beyond detuning: 10 years of progress and new challenges in the development and application of assays for HIV incidence estimation. AIDS. 2010 Oct 21.

7. Sakarovitch C, Rouet F, Murphy G, Minga AK, Alioum A, Dabis F, et al. Do tests devised to detect recent HIV-1 infection provide reliable estimates of incidence in Africa? J Acquir Immune Defic Syndr. 2007 May 1;45(1):115-22.

8. Marinda ET, Hargrove J, Preiser W, Slabbert H, van Zyl G, Levin J, et al. Significantly diminished long-term specificity of the BED capture enzyme immunoassay among patients with HIV-1 with very low CD4 counts and those on antiretroviral therapy. J Acquir Immune Defic Syndr. 2010 Apr 1;53(4):496-9.

9. Guy R, Gold J, García Calleja JM, Kim AA, Parekh B, Busch M, et al. Accuracy of serological assays for detection of recent infection with HIV and estimation of population incidence: a systematic review. Lancet Infect Dis. 2009;9(12):747-59.

10. Bärnighausen T, Wallrauch C, Welte A, McWalter TA, Mbizana N, Viljoen J, et al. HIV incidence in rural South Africa: comparison of estimates from longitudinal surveillance and cross-sectional cBED assay testing. PLoS ONE. 2008;3(11):e3640.

11. Hargrove JW, Humphrey JH, Mutasa K, Parekh BS, McDougal JS, Ntozini R, et al. Improved HIV-1 incidence estimates using the BED capture enzyme immunoassay. AIDS. 2008 Feb 19;22(4):511-8.

12. Karita E, Price M, Hunter E, Chomba E, Allen S, Fei L, et al. Investigating the utility of the HIV-1 BED capture enzyme immunoassay using cross-sectional and longitudinal seroconverter specimens from Africa. AIDS. 2007 Feb 19;21(4):403-8.

13. Todd J, Glynn JR, Marston M, Lutalo T, Biraro S, Mwita W, et al. Time from HIV seroconversion to death: a collaborative analysis of eight studies in six low and middle-income countries before highly active antiretroviral therapy. AIDS. 2007;21(Suppl 6):S55-S63.

14. Hallett TB, Gregson S, Gonese E, Mugurungi O, Garnett GP. Assessing evidence for behaviour change affecting the course of HIV epidemics: A new mathematical modelling approach and application to data from Zimbabwe Epidemics. 2009;1:108-17 (doi:10.1016/j.epidem.2009.03.001).

15. Hallett T. Monitoring HIV epidemics: declines in prevalence do not always mean good news. AIDS. 2009 Jan 2;23(1):131-2.

16. Bärnighausen T, Tanser F, Hallett T, Newell ML. Short communication: Prioritizing communities for HIV prevention in sub-Saharan Africa. AIDS Res Hum Retroviruses. 2010 Apr;26(4):401-5.

17. Lopman BA, Nyamukapa C, Hallett TB, Mushati P, Spark-du Preez N, Kurwa F, et al. Role of widows in the heterosexual transmission of HIV in Manicaland, Zimbabwe, 1998-2003. Sex Transm Infect. 2009 Apr;85 Suppl 1:i41-8.

18. Zaba B, Todd J, Biraro S, Shafer LA, Lutalo T, Ndyanabo A, et al., editors. Diverse age patterns of HIV incidence rates in Africa (TUAC0201) XVII International AIDS Conference; 2008 3-8 August 2008; Mexico City.

19. Landsberger HA. Hawthorne Revisited. Ithaca1958.

20. Padian NS, McCoy SI, Balkus JE, Wasserheit JN. Weighing the gold in the gold standard: challenges in HIV prevention research. AIDS. 2010 Mar 13;24(5):621-35.

21. Baeten JM, Richardson BA, Martin HL, Jr., Nyange PM, Lavreys L, Ngugi EN, et al. Trends in HIV-1 incidence in a cohort of prostitutes in Kenya: implications for HIV-1 vaccine efficacy trials. J Acquir Immune Defic Syndr. 2000 Aug 15;24(5):458-64.

22. White PJ, Ward H, Garnett GP. Is HIV out of control in the UK? An example of analysing patterns of HIV spreading using incidence-to-prevalence ratios. AIDS. 2006 Sep 11;20(14):1898-901.

23. Montaner JS, Lima VD, Barrios R, Yip B, Wood E, Kerr T, et al. Association of highly active antiretroviral therapy coverage, population viral load, and yearly new HIV diagnoses in British Columbia, Canada: a population-based study. Lancet. 2010 Aug 14;376(9740):532-9.

24. UNAIDS. What countries need: Investments needed for 2010 targets (<http://data.unaids.org/pub/Report/2009/JC1681_what_countries_need_en.pdf>, accessed 31 Oct, 2010)2010.

25. UNAIDS. Modes of Transmission Study - Guidelines for Country Teams (<http://www.unaidsrstesa.org/userfiles/file/Guidelines_for_the_Modes_of_Transmission_Project-Version12.pdf>, accessed 31-10-10). 2008.

26. Kontio K. Analysis of HIV Prevention Response and Modes of HIV Transmission Studies:

Outcome Evaluation Report (<http://www.unaidsrstesa.org/files/u1/Synthesis_outcome_evaluation_-_31_July09.pdf>, accessed 31-10-10)2009.

27. Parekh BS, Kennedy MS, Dobbs T, Pau CP, Byers R, Green T, et al. Quantitative detection of increasing HIV type 1 antibodies after seroconversion: a simple assay for detecting recent HIV infection and estimating incidence. AIDS Res Hum Retroviruses. 2002 Mar 1;18(4):295-307.

28. Bärnighausen T, McWalter TA, Rosner Z, Newell ML, Welte A. HIV incidence estimation using the BED capture enzyme immunoassay: systematic review and sensitivity analysis. Epidemiology. 2010 Sep;21(5):685-97.

29. Tuan N, Le L, Shah N, Kim A. Validation Study of HIV-1 Incidence Assays in Vietnam. The Second Asia Regional HIV Surveillance Meeting, Lessons Learned in Second Generation Surveillance: the Asia Experience; Ho Chi Minh City, Vietnam2010.

30. Kim AA, Hallett T, Stover J, Gouws E, Musinguzi J, Mureithi PK, et al. Estimates of HIV incidence among adults in Kenya and Uganda: a systematic comparison of multiple methods. 17th Conference on Retroviruses and Opportunistic Infections San Francisco, USA2010.

31. Hallett TB, Ghys P, Bärnighausen T, Yan P, Garnett GP. Errors in 'BED'-derived estimates of HIV incidence will vary by place, time and age. PLoS ONE. 2009;4(5):e5720.

32. Hladik W, Olara D, Were W, Mermin J, Downing R, editors. The Effect of Antiretroviral Treatment on the Specificity of the Serological BED HIV-1 Incidence Assay (Abstract 998). The 2007 HIV/AIDS PEPFAR Implementers' Meeting; 2007 June, 2007; Kigali, Rwanda.

33. McDougal JS, Parekh BS, Peterson ML, Branson BM, Dobbs T, Ackers M, et al. Comparison of HIV type 1 incidence observed during longitudinal follow-up with incidence estimated by cross-sectional analysis using the BED capture enzyme immunoassay. AIDS Res Hum Retroviruses. 2006 Oct;22(10):945-52.

34. McWalter TA, Welte A. Relating recent infection prevalence to incidence with a sub-population of assay non-progressors. J Math Biol. 2010 Jul 25;60(5):687-710.

35. Welte A, McWalter TA, Laeyendecker O, Hallett TB. Using tests for recent infection to estimate incidence: problems and prospects for HIV. Euro Surveill. 2010;15(24).

36. Sedia™. Sedia™ BED HIV-1 Incidence EIA. Enzyme Immunoassay for Population Estimates of HIV-1 Incidence [Cat. No. 1000] [package insert]. 2009.

37. Mermin J, Musinguzi J, Opio A, Kirungi W, Ekwaru JP, Hladik W, et al. Risk factors for recent HIV infection in Uganda. JAMA. 2008 Aug 6;300(5):540-9.

38. Todd J, Lutalo T, Kaleebu P. Estimating Incidence of HIV Infection in Uganda. JAMA. 2009 January 14, 2009;301(2):159-a-60.

39. Hallett T, Garnett G. Estimating Incidence of HIV Infection in Uganda. JAMA. 2009 January 14, 2009;301(2):159-.

40. Mermin J, Musinguzi J, Hladik W. Estimating Incidence of HIV Infection in Uganda--Reply. JAMA. 2009 January 14, 2009;301(2):160-a-1.

41. Brookmeyer R. Should biomarker estimates of HIV incidence be adjusted? AIDS. 2009 Feb 20;23(4):485-91.

42. Brookmeyer R. On the statistical accuracy of biomarker assays for HIV incidence. J Acquir Immune Defic Syndr. 2010 Aug 1;54(4):406-14.

43. Hargrove JW. BED estimates of HIV incidence must be adjusted. AIDS. 2009 Sep 24;23(15):2061-2; author reply 6-8.

44. Welte A, McWalter TA, Bärnighausen T. Reply to 'Should biomarker estimates of HIV incidence be adjusted?'. AIDS. 2009 Sep 24;23(15):2062-3; author reply 6-8.

45. McDougal JS. BED estimates of HIV incidence must be adjusted. AIDS. 2009 Sep 24;23(15):2064-5; author reply 6-8.

46. Thomas HI, Wilson S, O'Toole CM, Lister CM, Saeed AM, Watkins RP, et al. Differential maturation of avidity of IgG antibodies to gp41, p24 and p17 following infection with HIV-1. Clin Exp Immunol. 1996 Feb;103(2):185-91.

47. Chawla A, Murphy G, Donnelly C, Booth CL, Johnson M, Parry JV, et al. Human immunodeficiency virus (HIV) antibody avidity testing to identify recent infection in newly diagnosed HIV type 1 (HIV-1)-seropositive persons infected with diverse HIV-1 subtypes. J Clin Microbiol. 2007 Feb;45(2):415-20.

48. Suligoi B, Galli C, Massi M, Di Sora F, Sciandra M, Pezzotti P, et al. Precision and accuracy of a procedure for detecting recent human immunodeficiency virus infections by calculating the antibody avidity index by an automated immunoassay-based method. J Clin Microbiol. 2002 Nov;40(11):4015-20.

49. Wei X, Liu X, Dobbs T, Kuehl D, Nkengasong JN, Hu DJ, et al. Development of two avidity-based assays to detect recent HIV type 1 seroconversion using a multisubtype gp41 recombinant protein. AIDS Res Hum Retroviruses. 2010 Jan;26(1):61-71.

50. Masciotra S, Dobbs T, Candal D, Hanson D, Delaney K, Rudolph D, et al. Antibody avidity-based assay for identifying recent HIV-1 infections Based on Genetic Systems[TM] 1/2 Plus O EIA [#937]. Conference on Retroviruses and Opportunistic Infections; San Francisco2010.

51. Laeyendecker O, Oliver A, Astemborski J, Owen SM, Kirk G, Mehta S, et al. Improved Precision of Cross-Sectional HIV Incidence Testing Using a Multi-assay Algorithm that Includes BED and an Avidity Assay with Modified Assay Cut-offs [#935]. Conference on Retroviruses and Opportunistic Infections; San Francisco2010.

52. UNAIDS Reference Group on Estimates Modelling and Projections. Statement on the use of the BED-assay for the estimation of HIV-1 incidence for surveillance or epidemic monitoring. Wkly Epidemiol Rec. 2006;81(4):40-1.

53. Surveillance and Survey and the Laboratory Working Groups to the Office of the Global AIDS Coordinator. Intermin Recommendations For The Use Of The BED Capture Enzyme Immunoassay For Incidence Esimtation and Surveillance. (Available from: <http://www.cdc.gov/nchstp/od/GAP/docs/surveillance/Interim%20Recommendations%20for%20the%20use%20of%20the%20BED%20capture%20enzyme%20immunoassay%20for%20incidence%20estimation%20and%20surveillance%20Approved%20November%2021%202006%20(2).pdf)2006>.

54. Family Health International. Global Landscape & Market Assessments2009.

55. Curtis KA, Kennedy MS, Candal D, Hanson D, Delaney K, Charurat M, et al. Development of a Bead-based, Multiplex Assay for Estimation of Recent HIV-1 Infection. Conference on Retrovirus and Opportunistic Infection (CROI)2010.

56. Family Health International. Development of assays to estimate HIV incidence, Meeting Proceedings, Chapel Hill, NC. Proceedings of a meeting held May 13-14, 20009. North Carolina2009.

57. Parekh B, Hanson D, Hargrove J, Branson B, Green T, Dobbs T, et al. Determination of mean recency period for estimation of HIV-1 incidence with the BED-capture EIA in persons infected with diverse subtypes. AIDS Res Hum Retroviruses. In Press.

58. Kassanjee R, Welte A, McWalter TA, Viljoen J, Barnighausen T, Newell M-L, et al. Calibrationof BED assay for use in incidence estimation. 5th IAS Conference on HIV Pathogenesis, Treatment and Prevention; Cape Town, South Africa2009.

| **Figure 1: Uncertainty in Estimates of HIV incidence using incidence assay.** |
| --- |
| 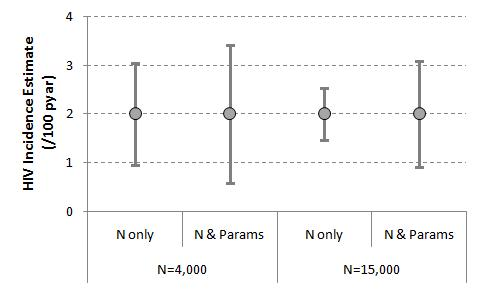 |
| Estimates of HIV incidence (circles) and associated 95% confidence interval (whiskers) for a hypothetical survey in a population with incidence rate of 2/100 person-years at risk (pyar). Estimates are shown for surveys with sample sizes (N) of 4,000 and 15,000. The width of the 95% confidence interval is compared when the calculations are based only on random sampling errors that are determined by survey sample size (‘N only’) with when the uncertainty in the characteristics of the incidence tests are also incorporated (‘N & Params’). The figure illustrates that large sample sizes are required to produce precise incidence estimates and that confidence intervals that are calculated without also incorporating uncertainty in parameter estimates suggest the estimate is much more precise than is actually the case. [Calculations follow method of McWalter & Welte ([34](#_ENREF_34)), and assume FRR=5%, incidence=2%/pyar, prevalence=20% and true coefficient of variation of parameter estimates = 15%]. |

| **Figure 2: Key milestones and decision points in the critical path over the next five years.** |
| --- |
|  |
| Anticipated timing of major milestones (numbered diamonds with solid lines) and key decisions (red circles with dashed lines) over the next five years in the critical path. |

| **Box 1: Who needs a reliable HIV incidence assay?** | | |
| --- | --- | --- |
| **Area** | **Function** | **Users** |
| ***Impact evaluation*** | To determine the impact of large-scale interventions on the rate of new infections. | Implementers;  Donors/funders;  Researchers;  Advocates. |
| ***Surveillance*** | To monitor transmission patterns, identify at-risk groups, and detect emerging trends in epidemic. | Ministries of Health;  Donors / funders;  Advocates. |
| ***Programme and resource planning*** | To optimally target interventions and to plan for future service requirements (e.g. treatment slots). | Programme planners;  Ministries of Health  Advocates. |
| ***Trials*** | To estimate pre-trial incidence to inform trial planning (required sample size, trial length, etc.), and as an end-point in community-based effectiveness trials, substantially reducing costs. | Clinical trial/research; organizations;  Funders/donors;  Researchers. |

| **Box 2: A Diagnostic Tool For Recent Infection** |
| --- |
| “Incidence Assays” are used to estimate the incidence rate in a population. “Diagnostic tools” for recent (or “acute”) infection are used to indicate whether or not a particular individual has been infected within a certain period of time. Reliable information about whether or not an infected individual was infected recently could provide evidence on where and among whom new infections are occurring, enhance partner notification and contact tracing interventions (to interrupt infectious spread), help guide the clinical management of patients, support HIV pathogenesis studies, and allow a scientific investigation of the contact network through which HIV is spreading. Given these applications, a more substantial market demand can be anticipated for an individual diagnostic test for recent infection than for population-based assays ([54](#_ENREF_54), [56](#_ENREF_56)).  However, these two applications of tests for recent HIV infection are *not* the same. One key difference between the characteristics of population-based incidence assays and clinical diagnostic tools is that an individual diagnostic requires the measurement of the target biomarker to have high *predictive value* for the individuals’ time since infection. With substantial between-individual variability in many biological targets, this presents a challenge. There is no such requirement for *population-based* incidence assays, however, for which only the *average* response in populations is important. For instance, if a population-based incidence assay like the BED assay has a mean duration of 197 days, this does *not* mean that a result above a certain threshold indicates a chronic (>197 days) infection *in that individual*; in fact, ~30% of individuals infected for 197 days would have a result below that threshold ([57](#_ENREF_57)).  The time scale over which population-based incidence assays and individual diagnostic tools need to be responsive is also different. Diagnostic tools need to detect individuals infected in the last few weeks or few months. However, this short duration would not be useful for incidence assays, because the sample sizes required to achieve a precise estimate of incidence (or to power a comparison of two incidence estimates) with such a marker would be prohibitively large. Tools used to detect individuals’ recentness of infection in surveillance application (such as analysing the behaviours of individuals with recent infections to infer current epidemic foci) still need high individual level predictive value but they may be useful if they indicate infection within a longer period that perhaps extends to 1-2 years depending the epidemiological situation.  Optimally, both purposes -- a population-based assay for incidence measurement, and an individual diagnostic assay of recent infection -- could be served by a single assay with a readout that holds high predictive value for the time since infection over a period of at least 4 months. The result of the assay could then be interpreted in one way for incidence estimation (using a particular threshold) and another for individual diagnosis (using a different threshold or another form of analysis). However, further work will be required to (i) better understand what information about an individual’s timing of infection is generated by assays with different performance characteristics, (ii) the extent to which this would be useful for clinicians and public health interventions under various scenarios. |

| **Box 3: Properties of an HIV Incidence Assay** |
| --- |
| *Incidence Test Metrics*  Incidence assays are used to estimate HIV incidence because they measure a well-characterized biomarker for the recentness of infection. In the ideal case the assay works so that one result (‘test-positive’) is returned for a period early in infection and at other times a different result (‘test-negative’) is returned. The average time it takes for individuals to progress from the ‘test-positive’ to the ‘test-negative’ state is called the ‘Mean duration’, $\omega$ (see figure). Incidence can thus be estimated as $I=\frac{N_{R+}/N_{U}}{\omega}$, where $N_{R+}$ is the number ‘test-positive’ and $N_{U}$is the number not infected, which is similar to the classic epidemiological calculation, “Prevalence = Incidence X Duration” ([34](#_ENREF_34), [35](#_ENREF_35)).  However, in practise, a subset of individuals in a population may not ever progress to the ‘test-negative’ stage and some individuals that had progressed to the test-negative state may later regress to the test-positive state. The proportion of chronically infected individuals with a ‘test positive’ result in a particular population at a particular time can be measured (among a representative sample of infected individuals known not to have been infected recently) and is termed the ‘false recent rate’ (FRR), $\varepsilon$ (see figure). Note that this parameter is not equivalent to the traditional definitions of test ‘specificity’ or ‘negative predictive value’.  The updated statistical estimator that allows an unbiased estimate of HIV incidence using an imperfect test (i.e. a false recent rate greater than zero) is $I=\frac{N_{R+}-\left(\frac{\varepsilon}{1-\varepsilon}\right)N_{R-}}{\omega N_{U}}$, where $N_{R-}$is the number ‘test-negative’ ([34](#_ENREF_34)). But this estimator only works if the test characteristics ($\varepsilon$ and $\omega$) are accurately and precisely measured for that particular population at that particular time (both these parameters can vary substantially between populations and over time ([6](#_ENREF_6), [9](#_ENREF_9), [30](#_ENREF_30), [58](#_ENREF_58))). If the estimate for these calibration parameters is not accurate, there will be substantial bias in the estimate of incidence ([10](#_ENREF_10), [11](#_ENREF_11), [31](#_ENREF_31)); and if they are not precisely estimated then incidence estimates will be uncertain even if the cross-sectional population survey is large. Critically, the mean duration and the false recent rate operate as a pair of parameters that jointly specify the test characteristics, so in a calculation, the way in which those parameters are evaluated must be the same.  For many applications of the first generation assays, these test characteristics have not been measured accurately for the population in which the test is being used and this is the reason that published assay-derived estimates have been inaccurate ([28](#_ENREF_28)).  **Figure:** Proportion of individuals ‘test positive’ on the incidence assay versus time since infection. The two main parameters (mean duration and FRR) are indicated under the simplifying assumption that individuals do not progress (this assumption is not made in the statistical equations but is made here only to simplify the graphic presentation). |

| **Box 4: A Target Product Profile For An “Incidence Assay or Algorithm”** |
| --- |
| A target product profile (TPP) lists key technical specifications that define a product that would be suitable for its intended use ([54](#_ENREF_54), [56](#_ENREF_56)). This list of product specifications is designed for manufacturers and product developers to guide development. The TPP should reflect, to the extent possible, a balance between the needs of users and technical feasibility: placing a bar for performance too high or too low could be detrimental to development and adoption of an assay. A TPP can also serve as a “straw man” argument to help guide discussions, and it will be important to continuously revise the TPP and the appropriate range of performance as development and evaluation progress.  Building on the investigation of the WHO working group, and together with several stakeholders, here we present the current TPP. This attempts to articulate more precisely our uniform desire for an *easy-to-use, cross-sectional, standardized assay that could be used routinely to reliably estimate incidence.* Extensive discussions with all the stakeholders guided which particular criteria will be included in the TPP and what range of performance is reasonable within the relevant timeline (2-3 years). These currently include: the ‘mean duration’ and FRR, the populations in which it can be used, “ease of use”, unit cost, algorithm, sample type, sample volume, infrastructure requirements, storage/shipping conditions, incubation temperature, shelf life, training and regulatory pathway.  Two of the most critical specifications in the TPP for an HIV incidence assay are the false recent rate (FRR) and mean duration (see Box 3). Smaller values of the FRR ($\varepsilon$) and larger values of the mean duration ($\omega$) will give more accurate and precise estimates of HIV incidence. The optimal values for the parameters would be $\varepsilon$=0% and $\omega$=12 months and for these values to be exactly the same for all clades, populations and epidemic types.  However, the minimum acceptable values of these specifications (that would ensure reliable point estimation of incidence, at least in high incidence settings such as southern Africa) are made by considering the robustness of the incidence estimate that could be generated by using an incidence assay, given logistical and statistical requirements. It should be noted that the minimum requirements will vary by application (estimates of absolute level of incidence, trend over time, or comparisons between population) and epidemic setting (incidence rate, incidence-to-prevalence ratio), and the considerations below only attempt to make useful broad statements across that range.  First, the data generated need to be suitable for robust statistical analysis. Basic mathematical assumptions that are inherent in all estimators require assay test characteristics and estimates of incidence to be approximately linearly related. Although in fact this is not the case for general assay characteristics and epidemiological contexts, estimates can still be safely made across a wide range of epidemiological conditions for an assay with a false recent rate confidently measured to be less than 2% in several different real populations (with different clades, epidemic types, treatment coverage etc). At FRR values higher than 2% there is also a rapid loss in statistical power and it becomes more likely that the FRR varies more substantially between populations and over time (increasing the risk of using an inaccurate FRR measured earlier or in a different population, potentially introducing large errors).  Further, if we also aim for an incidence assay that is of comparable statistical power to a 1-year cohort study, then a value of the mean duration between 4 and 12 months is sufficient. This range will ensure that incidence estimates using an assay are as *precise* as those estimated from a cohort study (as well as more representative for the reasons indicated in the main text). Although there is a modest (less than 25%) increase in the total number of test administered to generate incidence estimates compared to the cohort approach, this is considered acceptable because it would likely be offset by the lower cost of conducting a single cross-sectional survey.   \| **Specification** \| **Acceptable Performance** \| **Ideal Performance** \| \| --- \| --- \| --- \| \| **Intended Use** \| Population-based incidence estimate \| Population-based incidence estimate, prevention-trial planning, community-level prevention intervention studies \| \| **Target Population** \| Specific to clade \| All clades \| \| **False Recent Rate (FRR)** \| Confidently measured to be less than 2% in different populations (with different clades, epidemic phases, treatment coverage etc) \| 0% in all population (No evidence of false-recent classifications). \| \| **Mean Duration** \| 4 months (95% CI, +/- 0.2) \| 1 year (95% CI, +/- 0.2) \| \| **Algorithm** \| Included in a RITA \| None required \| \| **Analyte** \| Any \| Any \| \| **Sample Type** \| Frozen serum, frozen plasma \| Frozen serum or plasma , dried blood spots (or other easily obtained and stored sample) \| \| **Sample Volume** \| 1 mL \| 10 uL or fingerstick \| \| **Infrastructure requirements** \| Centralized laboratory facility (clean water and electricity available) \| None (all reagents and necessary materials to run assay are in self-contained kit) \| \| **Storage/Shipping Conditions** \| 4-25 °C \| Ambient temperature \| \| **Incubation Temperature** \| 4-25 °C \| Ambient temperature \| \| **Shelf Life** \| 9 months \| >18 months \| \| **Training** \| Laboratory technician can be proficient with one week’s training based on proficiency testing \| Minimal training would allow any health worker to conduct the assay \| \| **Regulatory Pathway** \| GMP or ISO 13485 or equivalent, and/or approval by national governing body \| FDA and equivalents \|   **Table:** Target Product Profile |

***Appendix:***

***Members of the Incidence* *Assay Critical Path Working Group***

| **Name** | **Organization** | **Email** |
| --- | --- | --- |
| Rifat Atun | Global Fund | Rifat.Atun@theglobalfund.org |
| David Burns | National Institutes of Health | [burnsda@niaid.nih.gov](mailto:burnsda@niaid.nih.gov) |
| Jesus Maria  Jesus Maria | World Health Organization | callejaj@who.int |
| Mike Busch | Blood Systems | [mbusch@bloodsystems.org](mailto:mbusch@bloodsystems.org) |
| Paul Constestable | Ortho-Clinical Diagnostics | [pcontest@its.jnj.com](mailto:pcontest@its.jnj.com) |
| Gina Dallabetta | Bill & Melinda Gates Foundation | [gina.dallabetta@gatesfoundation.org](mailto:gina.dallabetta@gatesfoundation.org) |
| Gonzalo Domingo | Program for Appropriate Technology in Health | [gdomingo@path.org](mailto:gdomingo@path.org) |
| Amb Eric Goosby | Office of the U.S. Global AIDS Coordinator | [GoosbyE@state.gov](mailto:GoosbyE@state.gov) |
| Patricia Garrett | SeraCare Life Sciences, Inc | [pgarrett@seracare.com](mailto:pgarrett@seracare.com) |
| Peter Ghys | UNAIDS | ghysp@unaids.org |
| Michael Gilbreath | National Institutes of Health | [gilbreathmj@niaid.nih.gov](mailto:gilbreathmj@niaid.nih.gov) |
| Tim Hallett | Imperial College London | timothy.hallett@imperial.ac.uk |
| Gottfried Hirnschall | World Health Organization | [hirnschallg@who.int](mailto:hirnschallg@who.int) |
| Charles Holmes | Office of the U.S. Global AIDS Coordinator | HolmesCB@state.gov |
| John Kaldor | University of New South Wales | [Jkaldor@nchecr.unsw.edu.au](mailto:Jkaldor@nchecr.unsw.edu.au) |
| Oliver Laeyendecker | Johns Hopkins University | olaeyen@jhmi.edu |
| [Tom McWalter](mailto:mcwalter@cam.wits.ac.za) | University of Witwatersrand | mcwalter@cam.wits.ac.za |
| Ronald Mink | Sedia | [rmink@sediabio.com](mailto:rmink@sediabio.com) |
| Gary Murphy | Health Protection Agency | [gary.murphy@hpa.org.uk](mailto:gary.murphy@hpa.org.uk) |
| Maurine Murtagh | Clinton Foundation | mmurtagh@clintonfoundation.org |
| Nancy Padian | Bill & Melinda Gates Foundation | nancy.padian@gmail.com |
| Peter Piot | Imperial College London | Peter.Piot@lshtm.ac.uk |
| Tom Quinn | Johns Hopkins University | [tquinn2@jhmi.edu](mailto:tquinn2@jhmi.edu) |
| Renee Ridzon | Bill & Melinda Gates Foundation | [renee.ridzon@gatesfoundation.org](mailto:renee.ridzon@gatesfoundation.org) |
| Bill Rodriguez | Bill & Melinda Gates Foundation | brod19@gmail.com |
| Christine Rousseau | Bill & Melinda Gates Foundation | christine.rousseau@gatesfoundation.org |
| Marco Schito | National Institutes of Health | [schitom@niaid.nih.gov](mailto:schitom@niaid.nih.gov) |
| Usha Sharma | National Institutes of Health | [USharma@niaid.nih.gov](mailto:USharma@niaid.nih.gov) |
| Alex Welte | South African Centre for Epidemiological Modelling and Analysis | alexwelte@sun.ac.za |
| Amy Wong | Clinton Foundation | [awong@clintonfoundation.org](mailto:awong@clintonfoundation.org) |

1. The terms ‘assays’ and ‘algorithms’ (combinations of assays) and ‘Recent Infection Testing Algorithms’ (RITA) are used interchangeably, unless otherwise stated. [↑](#footnote-ref-2)
2. Before additional screening using behavioural, immunologic, or virologic information. Note that the FRR can be defined crudely or after removal of known factors that lead to misclassification (in particular late stage of disease, ART). Whatever definition is used, it must match with the population data that is being analyzed and the population for which the mean duration parameter was estimated. [↑](#footnote-ref-3)
3. It should be noted that the ‘reference’ incidence estimates were often based on model approximation which will also carry substantial uncertainty, and that for populations with low incidence rate, small absolute differences between two incidence estimates generate large relative errors. [↑](#footnote-ref-4)
4. Calculation assumed mean duration of 185 days; http://www.sacema.com/page/assay-based-incidence-estimation [↑](#footnote-ref-5)
5. http://www.who.int/diagnostics_laboratory/links/hiv_incidence_assay/en/ [↑](#footnote-ref-6)
6. https://www.pepfar.net/C7/C8/HIV%20Incidence/default.aspx [↑](#footnote-ref-7)
7. Note that this does not refer to tests for reproducibility or congruence of tests results, but comparison of test characteristics relevant for estimation of incidence at population level. [↑](#footnote-ref-8)
